# Guidance Point Generation-Based Cooperative UGV Teleoperation in Unstructured Environment

**DOI:** 10.3390/s21072323

**Published:** 2021-03-26

**Authors:** Sen Zhu, Guangming Xiong, Huiyan Chen, Jianwei Gong

**Affiliations:** 1 Intelligent Vehicle Research Center, Beijing Institute of Technology, Beijing 100081, China; zhusen2000@bit.edu.cn (S.Z.); chen_h_y@263.net (H.C.); gongjianwei@bit.edu.cn (J.G.); 2 China North Vehicle Research Institute, Beijing 100072, China

**Keywords:** unmanned ground vehicle, human-machine cooperative control, unstructured environment, skeleton extraction, guidance point

## Abstract

Teleoperation is widely used for unmanned ground vehicle (UGV) navigation in military and civilian fields. However, the human operator has to limit speed to ensure the handling stability because of the low resolution of video, limited field of view and time delay in the control loop. In this paper, we propose a novel guidance point generation method that is well suited for human–machine cooperative UGV teleoperation in unstructured environments without a predefined goal position. The key novelty of this method is that the guidance points used for navigation can be generated with only the local perception information of the UGV. Firstly, the locally occupied grid map (OGM) was generated utilizing a probabilistic grid state description method, and converted into binary image to constructed the convex hull of obstacle area. Secondly, we proposed an improved thinning algorithm to extract skeletons of navigable regions from binary images, and find out the target skeleton related to the position of the UGV utilizing the k-nearest neighbor (kNN) algorithm. The target skeleton was reconstructed at the midline position of the navigable region using the decreasing gradient algorithm in order to obtain the appropriate skeleton end points for use as candidate guidance points. For visually presenting the driving trend of the UGV and convenient touch screen operation, we transformed guidance point selection into trajectory selection by generating the predicted trajectory correlative to candidate guidance points based on the differential equation of motion. Experimental results show that the proposed method significantly increases the speed of teleoperated UGV.

## 1. Introduction

Unmanned ground vehicles (UGV) have been very useful in a number of civilian and military fields, including rescue, reconnaissance and patrol mission. With the rapid development of sensor, communication and intelligent control, humans are able to increase their distance from dangerous tasks. Control of a UGV over a distance is referred to as teleoperation and has become a very popular control mode. While adding distance between the human operator and UGV can be advantageous to the safety of the human, it also introduces negative side effects. In particular, with this arrangement human operators often have to control the vehicle using 2D video feeds with low resolution and limited field of view [1]. To make matters more difficult, the video feed and control command are delayed hundreds of milliseconds because of data processing and transmission over a wireless network. Previous work has shown that time delays decrease the speed and accuracy with which human operators can complete a teleoperation task [2,3].

Many researchers have been working on methods to assist humans overcome these challenges inherent to teleoperation. For example, researchers have developed algorithms to combine camera and lidar data to generate 3D exocentric views for more intuitive teleoperation [4]. For well-defined tasks in structured environments, researchers could design integrated multi-sensor systems and developed algorithms to make the UGV more autonomous [5,6]. However, many UGVs carry out tasks in unstructured environments in which the current autonomous navigation technology is difficult to ensure the safety of vehicles, and still require human knowledge or expertise. Cosenzo and Barnes claim most military robotic systems will continue to require active human control or at least supervision with the ability to take over if necessary [7].

Some studies have suggested that human–machine cooperative control (versus manual control) of a UGV can improve task performance in high speed navigation [8]. Macharet and Florencio developed a method that uses artificial potential fields and vector field histograms for navigation with a human operator in the control loop [9]. Researchers in the automotive domain have conducted studies that show model predictive control (MPC) can be an effective tool in cooperative control. The MPC based methods augment driver inputs based on factors such as predicted vehicle stability [10] and predicted vehicle positions [11,12].

However, there are still gaps in the research and methods that attempt to best combine human operator and navigation control system of a UGV. More specifically, many of the previous works assumed that navigation control system knows the key guidance information of task. In many cases this is a reasonable assumption, e.g., an autonomous navigation task based on road network, it may be reasonable to assume that global path points have been determined in advance. In unstructured environments, e.g., off-road areas, often it is not reasonable to assume the navigation control system knows the human’s goal position.

This paper presents a novel guidance point generation method that is well suited for human–machine cooperative UGV teleoperation in unstructured environments without a predefined goal position. The key novelty of this method is that the guidance points used for navigation can be generated with only the local perception information of the UGV. Firstly, the locally occupied grid map (OGM) was generated utilizing a probabilistic grid state description method, and converted into binary image to constructed the convex hull of obstacle area. Secondly, we proposed an improved Zhang–Suen algorithm to extract skeletons of navigable regions from image, and find out the target skeleton related to the position of UGV utilizing the kNN algorithm. The target skeleton was reconstructed at the midline position of the navigable region using the decreasing gradient algorithm in order to obtain the appropriate skeleton end points for use as candidate guidance points. For convenient touch screen operation, we transformed guidance point selection into trajectory selection by generating the predicted trajectory correlative to candidate guidance points based on the differential equation of motion.

The remainder of this paper is organized as follows. Section 2 contains a review of work in the literature related to mainstream cooperative control techniques for UGV teleoperation. Section 3 introduces the proposed generating algorithm of candidate guidance points. Section 4 describes the human–machine cooperative control method base on guidance points selection. Section 5 introduces the hardware platform and the experimental setting, and analyses the experimental results in detail. Section 6 provides conclusions and suggestions for future research.

## 2. Related Works

With the continuous improvement of autonomy of UGVs, the relationship between the UGV and human operator has changed from the traditional master-slaver control to a more flexible cooperative relationship. In this paper the human–machine cooperative control refers in particular to the operator and the navigation control system being in the control loop at the same time, and sharing the task information, environmental information and UGV status to complete the driving task [13]. According to the differences of cooperative modes, the cooperative control can be divided into three categories: input correction cooperative control, haptic interaction cooperative control and guidance interaction cooperative control.

### 2.1. Input Correction Cooperative Control

**Figure 1:**
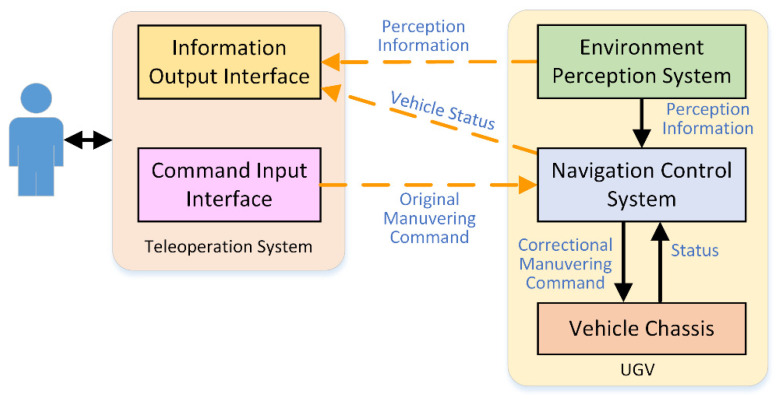
System overview of input correction cooperative control.

The navigation control system uses the control command generated by the autonomous navigation algorithm to modify the control command input by the operator through the human–machine interface, and transmits the results to the chassis controller, so as to realize cooperative control. Figure 1 shows an overview of the input correction cooperative control system.

Early research work was based on the assumption of fixed control ratio between operator and navigation control system to design the cooperative control method [14,15]. Reference [16] points out that the distribution of control right between human and machine should be adaptively adjusted according to the operator’s intention. Reference [17] uses the human factor model to predict the operator’s control behavior, and designs a nonlinear model prediction controller to assist the operator with minimal intervention. Some researchers believe that the operator’s freedom of control should be guaranteed in the process of cooperative control. Reference [18] proposed a human-machine input mixing method based on path homotropy concept, and introduced autonomous control action only when necessary to avoid collision, so as to achieve a balance between human freedom of operation and driving safety.

### 2.2. Haptic Interaction Cooperative Control

**Figure 2:**
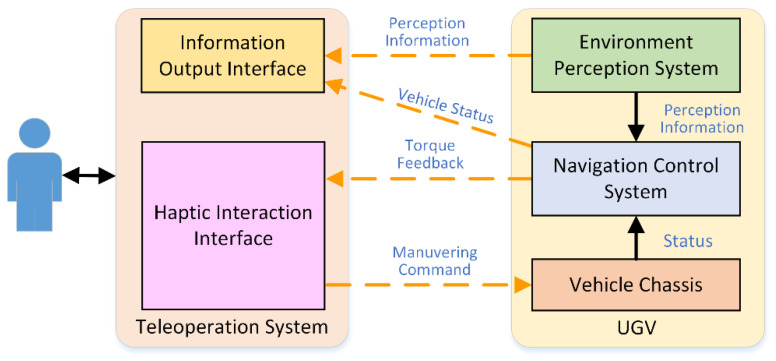
System overview of haptic interaction cooperative control.

A haptic interaction cooperative control mode has two advantages over input correction cooperative control method: (1) the operator continuously interacts with the UGV through haptic interface, and thus integrates into the control loop deeply; (2) this mode gives the operator higher control authority, and the operator can overcome the torque feedback from the navigation control system to take over the UGV completely [11]. Figure 2 shows an overview of the haptic interaction cooperative control system.

In reference [19], a haptic guidance with predictive capability was added to the cooperative control, which increases the completion rate of the remote driving task and the operator’s workload were improved to a certain extent. Reference [20] has proved that the performance of operators under cooperative control can be improved in both lane-keeping and vehicle-following tasks.

The key factor of haptic interaction cooperative control is to determine the desired auxiliary torque that conforms to the characteristics of human muscular motion. Reference [21] proves that the appropriate relationship model between muscular movement and expected torque is a key factor to improve the interaction performance in the process of cooperative control. Reference [22] studied haptic interaction cooperative control under different degrees of haptic feedback force, and found that the performance of the system improved more significantly when the feedback force was small. Reference [23] designed a dynamic cooperative steering control system by modeling the biomechanical impedance of the human arm, and realized dynamic torque feedback to the operator by adjusting the cooperative impedance.

### 2.3. Guidance Interaction Cooperative Control

**Figure 3:**
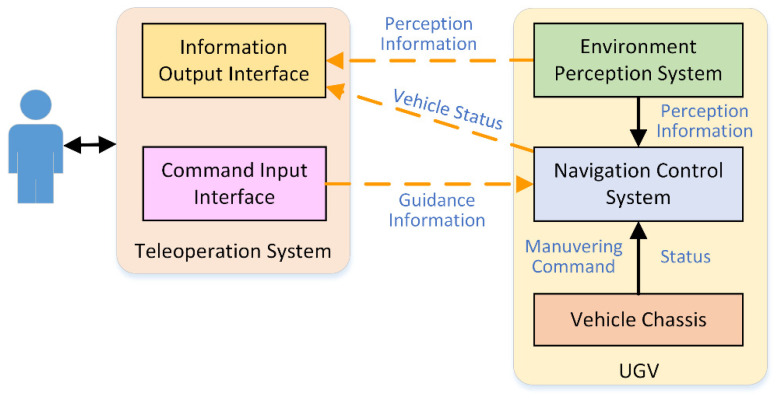
System overview of guidance interaction cooperative control.

Under the guidance interactive cooperative control mode, there is a hierarchical cooperative relationship between the operator and the navigation control system [24]. The operator makes a control decision according to the statues and environmental perception information from the UGV, and input corresponding global or local guidance information to navigate the UGV maneuvering toward target position. Figure 3 shows an overview of the guidance interactive cooperative control system.

Guidance control based on global path is a cooperative control method for wide range navigation. The operator plans the path based on the global map, and UGV takes the path as the guide to complete the maneuvering by relying on its own autonomous navigation ability [25]. In the process of autonomous maneuver, the operator can modify the global path at any time so as to realize the dynamic update of global guidance information [26].

In the local guidance control mode, the operator plans the local path based on the local environmental map to guide the UGV maneuvering in a limited area [27]. The key points guidance mode is also known as the “point-to-go” mode [28], which can be regarded as a special form of local guidance control. In this mode the operator selects a key point from the local map or the video image feedback from the UGV as a maneuvering target point.

For navigation control in an unstructured environment, the above guidance control methods have the following limitations:

1. Not all regions have high precision maps for global path guidance. Even for an area with a global map, due to the lack of timely update of map data, the seemingly passable area on the map is actually impassable when the natural environment changes, which leads to the infeasibility of the planned global path.
2. Due to the limitation of autonomous capability of a UGV, it is difficult to complete the task only by its own autonomous navigation system in the complex unstructured environment.
3. The operator needs to actively determine the position of the guidance point in the absence of decision support, which results in a large workload for the operator. On the other hand, if the guidance point is not entered in time, it will bring serious fluctuation of speed.

In view of these problems, the paper proposes a novel guidance point generation method that is well suited for human–machine cooperative UGV teleoperation in unstructured environments without a predefined goal position. The candidate points can be generated automatically with only the local perception information of the UGV. The operator selects a candidate point as the guidance points through the human-machine interface according to the driving intention. Subsequently, the navigation control system of the UGV generates feasible trajectory line according to its own motion state and environmental perception information, which can be tracked by the actuator.

## 3. Guidance Point Generation Method

The proposed generation method of candidate guidance points mainly includes the following three parts of work: (1) the generation of OGM; (2) obstacle description based on mathematical morphology; (3) generation of candidate guidance points based on skeleton extraction. The details will be covered in this section.

### 3.1. Generation of Occupied Grid Map (OGM)

**Figure 4:**
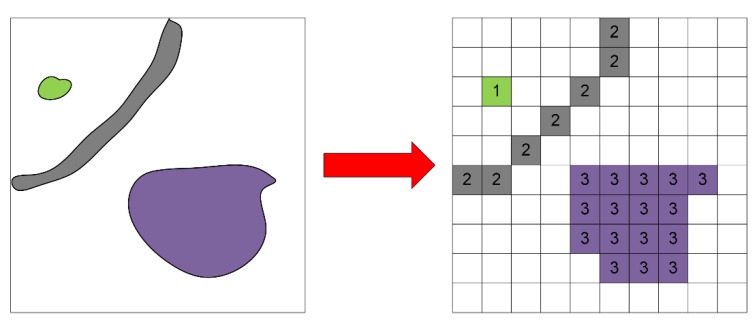
Occupied grid map.

In the field of environment modeling for UGV, the common environmental representation methods include geometric feature map, point cloud map and occupied grid map. The geometric feature map describes the object mainly using simple geometrical elements, e.g., points, lines and planes. This representation method can describe the scene well in the structured environment, but it is difficult to describe complex terrain accurately in the unstructured environment. As an intuitive representation of lidar measurement data, point cloud map contains all the information of the real world, but due to the large amount of data, it is not easy to store and manage. OGM divides the environment into grids, as shown in Figure 4. Each grid is given a probability of being occupied by an obstacle, which can simply represent the distribution of obstacles in the environment.

#### 3.1.1. Assumption

This paper uses lidar to collect environmental information in order to avoid the impact of changes in lighting and weather conditions on environmental features, and describes the surrounding environment of the UGV by OGM. Because the application scenario of the UGV is an unstructured outdoor environment, we can make the following assumptions:

- Assumption 1: The trajectory of the UGV is located on a two-dimensional plane to ensure that the measured data obtained at different times are in the same plane, so that the environmental information obtained by light detection and ranging (lidar) at close times contains the same environmental objects.
- Assumption 2: It is assumed that the environment is static, that is, the positions of all or most of the objects or features in the environment do not change over time. This assumption, also known as the Markov hypothesis, states that if the current state is known, the future state is independent of the past states.
- Assumption 3: Based on the assumption of two-dimensional plane motion of UGV, the 6 degrees of freedom (DOF) spatial motion of the UGV can be simplified into 3-DOF plane motion. The motion state of the UGV can be determined by its heading and 2-DOF position information.
- Assumption 4: It is assumed that the UGV is a rigid body during motion, and the influence of suspension changes, tire deformation and other factors on the vehicle body and the pitch and roll angle of the lidar is ignored, so as to improve the real-time performance of the map generation algorithm and meet the requirements of high-speed driving.

#### 3.1.2. OGM Update

We used a 325 × 150 OGM with a side length of 0.2 m, which corresponded to a range of 65 m × 30 m in the real environment. In the traditional binary OGM representation method, each grid only contains two states of “occupied” or “not occupied”, which are represented by 0 and 1 respectively. However, the obstacle detection algorithm based on lidar point cloud data faces the problem of missed detection and misdetection, which could lead to the incorrect state of some grids. In order to avoid this problem, a probabilistic grid state description method is adopted in this paper, and the probability range of grid occupied is [0, 1]. The occupancy state of a grid m can be expressed by a number p(m)∈[0, 1]. The p(m) is defined as:

(1) $$p\left(m\right)\{\begin{array}{l} >0.5\\ =0.5\\ <0.5 \end{array},$$

where p(m)>0.5 indicates that the grid is occupied by obstacles; p(m)<0.5 indicates that there is no obstacle in the grid; p(m)=0.5 indicates that the area where the grid is located has not been explored. At the initial moment of OGM creation, the states of all the grids in the map can be initialized to p(m)=0.5 since no valid observations of the environment have been made. Considering that the grids in the map are independent of each other, we only need to update the state of each grid to complete the update of the entire map. The probability expression of the grid occupied state is $p\left(m_{t}|x_{1:t},z_{1:t}\right)$. According to Bayes’ theorem, this expression can be further transformed into Equation (2).

(2) $$p\left(m_{t}|x_{1:t},z_{1:t}\right)=\frac{p\left(m_{t}|x_{1:t},z_{1:t-1}\right)\cdot p\left(z_{t}|x_{1:t},z_{1:t-1},m_{t}\right)}{p\left(z_{t}|x_{1:t},z_{1:t-1}\right)},$$

where t represents the time of data collection; $x_{1:t}$ and $z_{1:t}$ represent the position sequence of UGV and data series collected by lidar from the beginning of OGM creation (t=1) to the current time, respectively.

Since the grid state $m_{t}$ at time t is only related to the data series $z_{1:t}$, and the data $z_{t}$ have not been obtained at time t−1, the following equation can be obtained:

(3) $$p\left(m_{t}|x_{1:t},z_{1:t-1}\right)=p\left(m_{t-1}|x_{1:t-1},z_{1:t-1}\right).$$

Since environmental observation results $z_{t}$ at time t is only related to the position of UGV at time t and the grid state $m_{t-1}$ at time t−1, the following equation can be obtained:

(4) $$p\left(z_{t}|x_{1:t},z_{1:t-1},\;m_{t}\right)=p\left(z_{t}|x_{t},m_{t-1}\right),$$

By substituting Equations (3) and (4) into Equation (2), the simplified probability expression of a grid is obtained by using the following equation:

(5) $$p\left(m_{t}|x_{1:t},z_{1:t}\right)=\frac{p\left(m_{t-1}|x_{1:t-1},z_{1:t-1}\right)\cdot p\left(z_{t}|x_{t},m_{t-1}\right)}{p\left(z_{t}|x_{1:t},z_{1:t-1}\right)}.$$

According to Bayes’ theorem and the conditional independence principle, $p\left(z_{t}|x_{t},m_{t-1}\right)$ can be transformed as follows:

(6) $$p\left(z_{t}|x_{t},m_{t-1}\right)=\frac{p\left(z_{t}|x_{t}\right)\cdot p\left(m_{t-1}|x_{t},z_{t}\right)}{p\left(m_{t-1}\right)}.$$

By substituting Equation (6) into Equation (5), the formula for calculating the gird occupancy probability is defined as follows:

(7) $$p\left(m_{t}|x_{1:t},z_{1:t}\right)=\frac{p\left(m_{t-1}|x_{1:t-1},z_{1:t-1}\right)\cdot p\left(z_{t}|x_{t}\right)\cdot p\left(m_{t-1}|x_{t},z_{t}\right)}{p\left(z_{t}|x_{1:t},z_{1:t-1}\right)\cdot p\left(m_{t-1}\right)}.$$

Correspondingly, the formula for calculating the non-occupancy probability can also be obtained as follows:

(8) $$p\left({\bar{m}}_{t}|x_{1:t},z_{1:t}\right)=\frac{p\left({\bar{m}}_{t-1}|x_{1:t-1},z_{1:t-1}\right)\cdot p\left(z_{t}|x_{t}\right)\cdot p\left({\bar{m}}_{t-1}|x_{t},z_{t}\right)}{p\left(z_{t}|x_{1:t},z_{1:t-1}\right)\cdot p\left({\bar{m}}_{t-1}\right)}.$$

The left and right sides of Equation (7) divided by the left and right sides of Equation (8) gives us Equation (9).

(9) $$\frac{p\left(m_{t}|x_{1:t},z_{1:t}\right)}{p\left({\bar{m}}_{t}|x_{1:t},z_{1:t}\right)}=\frac{p\left(m_{t-1}|x_{1:t-1},z_{1:t-1}\right)\cdot p\left({\bar{m}}_{t-1}\right)\cdot p\left(m_{t-1}|x_{t},z_{t}\right)}{p\left({\bar{m}}_{t-1}|x_{1:t-1},z_{1:t-1}\right)\cdot p\left(m_{t-1}\right)\cdot p\left({\bar{m}}_{t-1}|x_{t},z_{t}\right)}.$$

The sum of probabilities of occupancy and non-occupancy of each grid is equal to 1 under any conditions, so we can transform Equation (9) to Equation (10):

(10) $$\frac{p\left(m_{t}|x_{1:t},z_{1:t}\right)}{1-p\left(m_{t}|x_{1:t},z_{1:t}\right)}=\frac{p\left(m_{t-1}|x_{1:t-1},z_{1:t-1}\right)\cdot \left(1-p\left(m_{t-1}\right)\right)\cdot p\left(m_{t-1}|x_{t},z_{t}\right)}{\left(1-p\left(m_{t-1}|x_{1:t-1},z_{1:t-1}\right)\right)\cdot p\left(m_{t-1}\right)\cdot \left(1-p\left(m_{t-1}|x_{t},z_{t}\right)\right)},$$

where $p\left(m_{t-1}|x_{1:t-1},z_{1:t-1}\right)$ represents the grid state at time t−1, and $p\left(m_{t-1}\right)$ is the prior value of the gird state, which is only related to the initial state of the grid. Based on the definition of the grid initial state $p\left(m_{t-1}\right)=0.5$, we simplify Equation (10) as follows:

(11) $$\frac{p\left(m_{t}|x_{1:t},z_{1:t}\right)}{1-p\left(m_{t}|x_{1:t},z_{1:t}\right)}=\frac{p\left(m_{t-1}|x_{1:t-1},z_{1:t-1}\right)\cdot p\left(m_{t-1}|x_{t},z_{t}\right)}{\left(1-p\left(m_{t-1}|x_{1:t-1},z_{1:t-1}\right)\right)\cdot \left(1-p\left(m_{t-1}|x_{t},z_{t}\right)\right)},$$

then the relation between $p\left(m_{t}|x_{1:t},z_{1:t}\right)$ and $p\left(m_{t-1}|x_{1:t-1},z_{1:t-1}\right)$ is obtained by using the following equations:

(12) $$p\left(m_{t}|x_{1:t},z_{1:t}\right)=\frac{S}{1+S},$$

(13) $$S=\frac{p\left(m_{t}|x_{t},z_{t}\right)}{1-p\left(m_{t}|x_{t},z_{t}\right)}\cdot \frac{p\left(m_{t-1}|x_{1:t-1},z_{1:t-1}\right)}{1-p\left(m_{t-1}|x_{1:t-1},z_{1:t-1}\right)}.$$

In Equation (13), $p\left(m_{t}|x_{t},z_{t}\right)$ represent estimated grid state based on $x_{t}$ and $z_{t}$, which also known as the inverse sensor model [29]. This model describes how to estimate the environment’s state based on the sensor observation position $x_{t}$ and the sensor measurement value $z_{t}$.

The light source of the lidar used in this paper is 905 nm infrared light, which has the advantages of good directivity and no penetration to most materials. Therefore, it can be considered that obstacles exist only at the location of the laser measurement point. However, due to the limitation of the accuracy of the lidar system, the reflection characteristics of the measured object and the difference of the transmission medium of the beam, the measurement data of the lidar may produce errors. For the above reasons, the possibility of inaccurate measurement is expressed in probability form based on the inverse sensor model.

(14) $$p\left(m_{t}|x_{t},z_{t}\right)=\{\begin{array}{ll} p_{emp} & if\;d>z_{t}\\ p_{occ} & if\;d=z_{t}\\ p_{unknown} & if\;d<z_{t} \end{array},$$

where:

- d is the distance between the center position of the grid and the lidar;
- $\;p_{emp}$ represent the occupancy probability of grid which locate in the area that the laser beam passes through, and $\;p_{emp}\neq 0$;
- $\;p_{occ}$ is the occupancy probability of grid where the measurement points are located, and $\;p_{occ}\neq 0$;
- $\;p_{unknown}$ is the occupancy probability of grid outside the measurement range, and $\;p_{unknown}=0.5$;

In the course of research it was found that objects with smaller diameters, such as tree trunks and shrubs, are observed less often than they are not. Theoretically we should set $\;p_{occ}+\;p_{emp}=1$, but if we follow this definition, the occupancy probability of grid where objects with smaller diameters are located will be less than 0.5 in most cases. This means that the locations of these obstacles will not be marked on the OGM. In order to facilitate the detection of fine objects in the environment, we set $p_{occ}+\;p_{emp}>1$ in practical application, where $p_{occ}=0.8$, $p_{emp}=0.4$.

#### 3.1.3. Binary Image Representation of OGM

**Figure 5:**
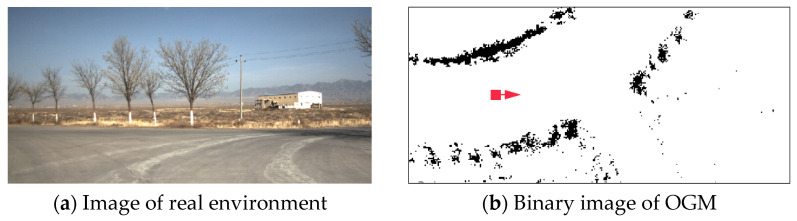
A binary image corresponding to a real environment.

To achieve skeleton extraction based on mathematical morphology, OGM needs to be transformed into binary images. Let each grid of OGM correspond to a pixel in the image, then the binary image is defined as:

(15) $$D\left(x,y\right)=\{\begin{array}{ll} 255 & if\;P\left(x,y\right)>T\\ 0 & otherwise \end{array},$$

where D(x,y) represents pixel gray value, and threshold T=0.5. Figure 5 shows a binary image corresponding to a real environment, where the black pixel represents the obstacle area, the white pixel represents the passable area, the red square indicates the position of the UGV in the diagram.

### 3.2. Obstacle Description Based on Mathematical Morphology

#### 3.2.1. Basic Operations of Mathematical Morphology

Mathematical morphology is a tool for extracting image components that are useful for representation and description [30]. The basic morphological operators are erosion, dilation, opening and closing. Let E be a Euclidean space or an integer grid, and A a binary image in E. The erosion of the binary image A by the structuring element B is defined by:

(16) $$A⊖B=\left\{z\in E\;|\;B_{z}\subseteq A\right\},$$

where $B_{z}$ is the translation of B by the vector z, i.e., $B_{z}=\left\{b+z\;|\;b\in B\right\},\;\forall_{z}\in E$.

The dilation of A by the structuring element B is defined by:

(17) $$A⊕B=\left\{z\in E|{\left(B^{s}\right)}_{z}\cap A\neq \phi \right\},$$

where $B^{s}$ denotes the symmetric of B, that is, $B^{s}=\left\{x\in E\;|-x\in B\right\}$.

The opening of A by B is obtained by the erosion of A by B, followed by dilation of the resulting image by B:

(18) A∘B=(A⊖B)⊕B.

The closing of A by B is obtained by the dilation of A by B, followed by erosion of the resulting image by B:

(19) A·B=(A⊕B)⊖B.

#### 3.2.2. Expanding of Obstacles

**Figure 6:**
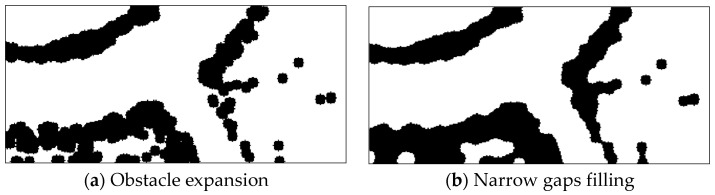
Obstacle description in binary image.

In order to treat the UGV as a particle in path planning, obstacles in binary image need to be expanded. Considering the irregular and discontinuous shape of obstacles in the unstructured environment, as well as the isolated distribution of some small obstacles in space, a disc-shaped structural element is selected for the expanding of the original image, and the radius of structural element is defined by the 1/2 width of the UGV. In this paper, the widths of the UGV and grid of OGM are 1.97 m and 0.2 m. Thus, the disc-shaped structural element has a radius of 5 pixels. The result of the obstacle expansion is shown below. As can be seen from the Figure 6a, there are narrow gaps between some obstacle areas. When we use the skeleton extraction method to generate the local path, it will produce some undesired paths which pass through narrow gaps. Although theoretically the UGV can safely pass through these narrow gaps, considering the positioning accuracy and control accuracy, there is still a risk of the UGV colliding with an obstacle. Therefore, we use the closing operator to fill the narrow gaps in the expanded binary image. The result is shown in Figure 6b.

### 3.3. Generating Algorithm of Candidate Guidance Points

A skeleton line is one of the important features to describe the plane region. It reflects the geometric characteristics and topological structure of the plane region. Skeleton extraction algorithms can be divided into three categories. The first is the topology thinning algorithms [31], the basic idea of which is to continuously strip the boundary points of the object in an iterative way until there is no extra boundary to strip off, and what remains is the skeleton. This method can ensure the connectivity of the skeleton, but it is sensitive to boundary noise, and the end of the skeleton may deviate from the center of the object. The second class of method is based on distance transformation [32], which forms the skeleton by looking for gradient ridges of the distance field. The position of the skeleton obtained by this method is accurate, but it is difficult to guarantee the connectivity of the skeleton. The third class of method is based on the Voronoi diagram [33], which has high complexity and requires pruning of the generated skeleton.

In order to generate the single-pixel skeleton with accurate connectivity in real time, this paper proposed an improved Zhang–Suen topology thinning algorithm to extract the skeleton [34]. In addition, we perform convex hull transformation on the obstacle area to solve the problem that the skeleton shape is sensitive to the object boundary noise. Furthermore, a solution based on the gradient descent method is given to improve the deviation problem of the skeleton end.

#### 3.3.1. An Improved Zhang–Suen Topology Thinning Algorithm

**Figure 7:**
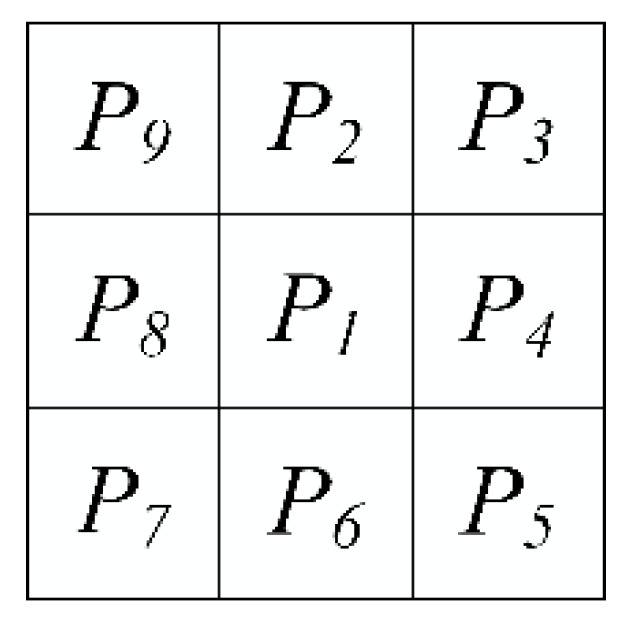
The neighbor pixels set of pixel P_1_.

The algorithm principle of Zhang–Suen topology thinning algorithm is as follows:

- Define the foreground pixel value of a binary image to be 1 and the background point pixel value to be 0. For a certain foreground pixel P1, the pixel set of its eight neighborhoods is shown in Figure 7, where P2~P9 are adjacent pixels of pixel P1;
- $M\left(P_{1}\right)=P_{2}+P_{3}+P_{4}+P_{5}+P_{6}+P_{7}+P_{8}+P_{9}$;
- $N\left(P_{1}\right)$ is defined as the number of times that the pixel value changes from 1 to 0 by traversing neighbor pixels around $P_{1}$ clockwise from $P_{2}$;
- The two steps are repeated until no pixels are deleted in either step, and the output is the thinning skeleton of the foreground in binary image.

**Step 1**: Iterate through all the pixels in the foreground, deleting the pixels that meet one of the following conditions.

(1) $2\leq M\left(P_{1}\right)\leq 6$;
(2) $N\left(P_{1}\right)=1$;
(3) $P_{2}\cdot P_{4}\cdot P_{6}=0$;
(4) $P_{4}\cdot P_{6}\cdot P_{8}\cdot =0$.

**Step 2**: Iterate through all the pixels in the foreground, deleting the pixels that meet one of the following conditions.

(1) $2\leq M\left(P_{1}\right)\leq 6$;
(2) $N\left(P_{1}\right)=1$;
(3) $P_{2}\cdot P_{4}\cdot P_{8}=0$;
(4) $P_{2}\cdot P_{6}\cdot P_{8}\cdot =0$.

**Figure 8:**
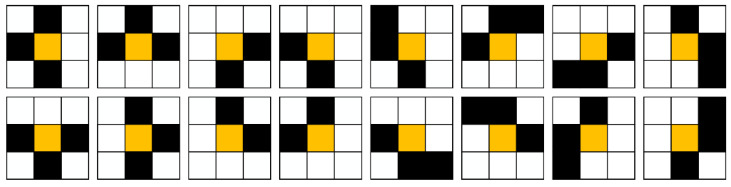
Non-single pixel skeletons generated by Zhang–Suen algorithm.

Although the Zhang–Suen algorithm has good real-time performance and can guarantee the connectivity of the skeleton, non-single pixel skeleton will appear at the fork, corner and step of skeleton. As shown in Figure 8, the yellow grid represents redundant pixels that need to be removed.

To solve the above problems, this paper proposes five criteria to identify redundant pixels:

(1) $\left(P_{2}\times P_{8}=1\right)\wedge \left(P_{4}+P_{5}+P_{6}+P_{9}=0\right)$;
(2) $\left(P_{6}\times P_{8}=1\right)\wedge \left(P_{2}+P_{3}+P_{4}+P_{7}=0\right);$
(3) $\left(P_{2}\times P_{4}=1\right)\wedge \left(P_{3}+P_{6}+P_{7}+P_{8}=0\right)$;
(4) $\left(P_{4}\times P_{6}=1\right)\wedge \left(P_{2}+P_{5}+P_{8}+P_{9}=0\right)$;
(5) $\left(P_{3}+P_{5}+P_{7}+P_{9}=0\right)\wedge \left(P_{2}+P_{4}+P_{6}+P_{8}=0\right)$.

**Figure 9:**
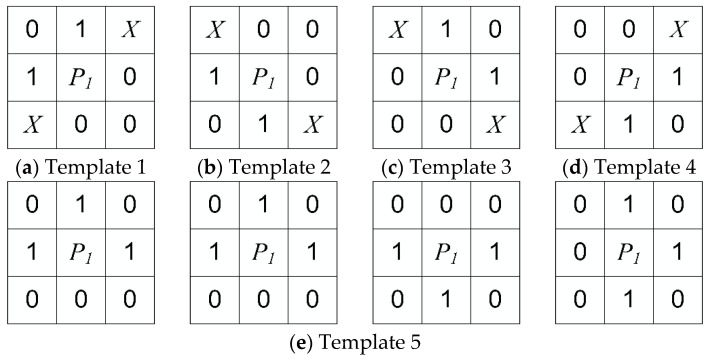
The templates for removing redundant pixels.

The first four criteria are used to identify redundant pixels at the fork and corner of skeleton. The last criterion is used to identify redundant pixels at the step of the skeleton. In order to facilitate binary image processing, we built five templates for eliminating redundant pixels based on the above criteria, as shown in Figure 9.

**Figure 10:**
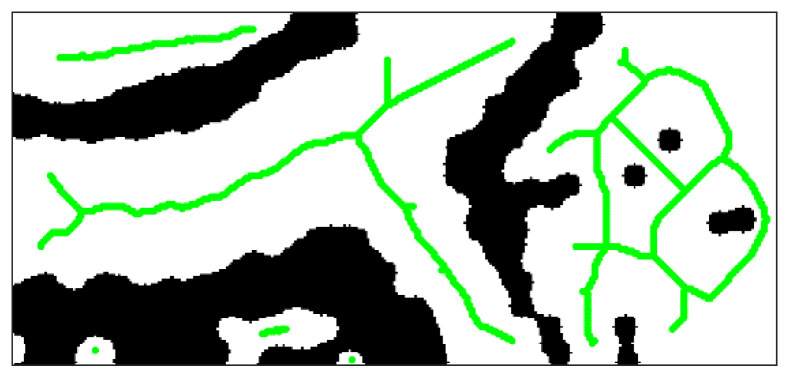
Skeletons of navigable regions.

Figure 10 shows extracted single-pixel skeletons of navigable regions by using an improved Zhang–Suen algorithm.

#### 3.3.2. Smooth Skeleton Generation Based on Convex Hull Transform

**Figure 11:**
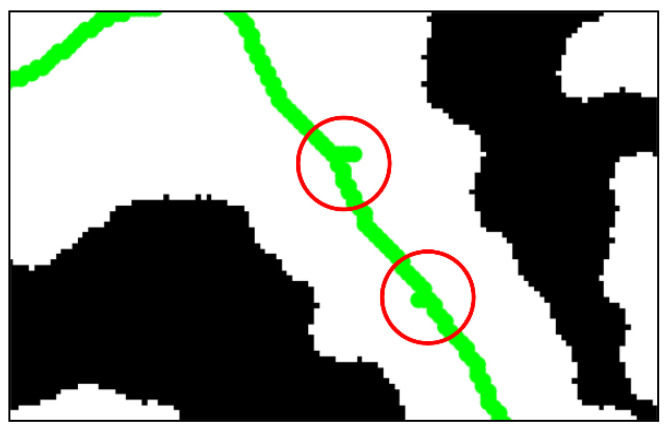
Spurious branches of skeleton.

Another disadvantage of the Zhang–Suen algorithm is that it is sensitive to boundary noise, which means that when the outline of navigable region is not smooth enough, the extracted skeleton is also not smooth and it is possible to generate spurious branches, as shown in red circle in Figure 11.

**Figure 12:**
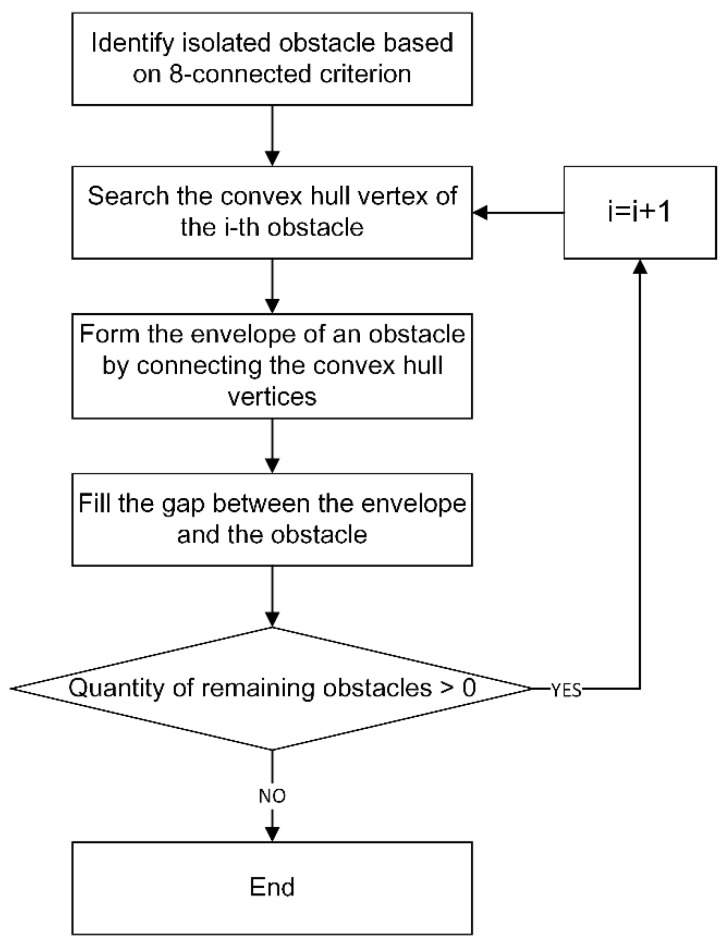
Flow chart of convex hull transform for grid map.

In this research, the obstacle convex hull is constructed to obtain the navigable region with smooth outline. The flow chart in Figure 12 describes the detail of convex hull transform for the grid map.

For obstacles based on grid representation, the internal occupied grid has no contribution to generating a convex hull, so it can be considered to eliminate the internal occupied grids as much as possible to improve the search speed of convex hull vertices. In this paper, a grid elimination method for irregular regions is proposed. This method consists of two phases:

**Phase 1:**

(1) The leftmost and rightmost grids of each row in the obstacle area are marked as unremovable;
(2) The grids at the top and bottom of each column in the obstacle area are marked as unremovable;
(3) Eliminate the remaining unmarked obstacle grids in the obstacle area.

**Figure 13:**
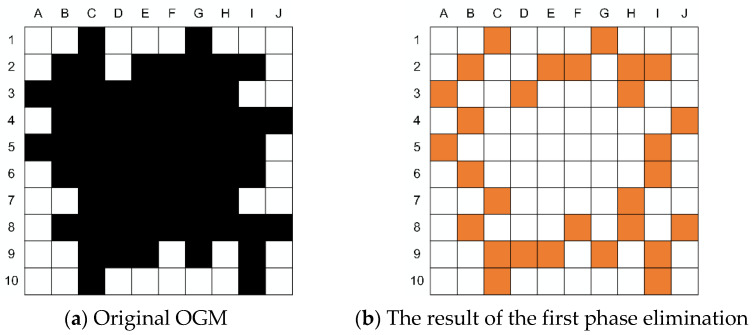

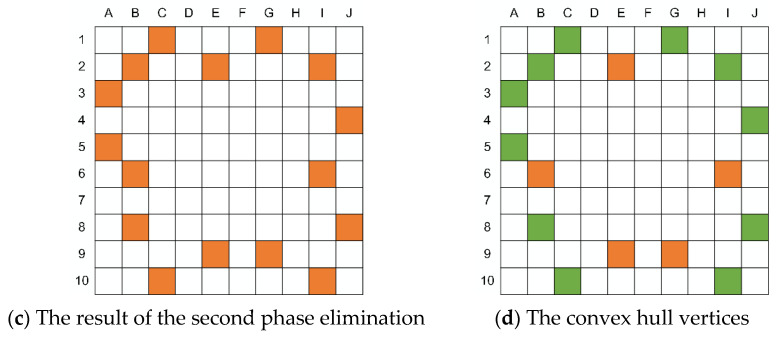
An example of convex hull vertices searching for irregular region.

From the result of the first phase elimination in Figure 13b we can know that there are still a lot of occupied grids inside the obstacle, which can be further removed in Phase 2.

**Phase 2:**

(1) Traverse the leftmost grid $L_{i}$ of each row. If the leftmost grid $L_{i-1}$ and $L_{i+1}$ of the upper and lower two rows are both on the left of $L_{i}$, or either is on the left of $L_{i}$, and the other is in the same column as $L_{i}$;
(2) Traverse the rightmost grid $R_{i}$ of each row. If the rightmost grid $R_{i-1}$ and $R_{i+1}$ of the upper and lower two rows are both on the right of $R_{i}$, or either is on the right of $R_{i}$, and the other is in the same column as $R_{i}$;
(3) Traverse the top grid $T_{i}$ of each column. If the top grid $T_{i-1}$ and $T_{i+1}$ of the left and right two columns are both on the top of $T_{i}$, or either is on the top of $T_{i}$, and the other is in the same row as $T_{i}$;
(4) Traverse the bottom grid $B_{i}$ of each column. If the bottom grid $B_{i-1}$ and $B_{i+1}$ of the left and right two columns are both on the below of $B_{i}$, or either is on the below of $T_{i}$, and the other is in the same row as $B_{i}$.

Compared with the result of the first phase, 14 occupied grids such as F2, H2, D3, H3, B4, I5, C7, H7, F8, H8, C9, D9 and I9 were further removed in the second stage, as shown in Figure 13c. Subsequently, the Graham scanning method [35] was used to search the convex hull vertices of obstacles in the remaining occupied grids. The search results are represented in green grids in Figure 13d.

**Figure 14:**
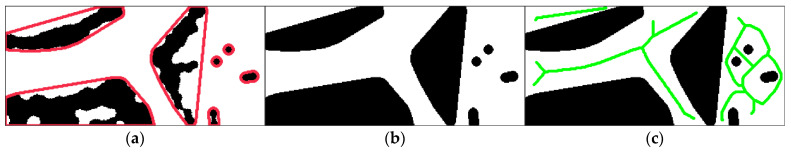
Smooth skeleton extraction for binary-image. (**a**) The red closed curves represent the envelopes of the obstacles; (**b**) result of convex hull transform; (**c**) extracted smooth skeletons after convex hull transform of obstacles.

After finishing the search of convex hull vertices, it is necessary to connect the vertices with straight lines to form an envelope. Since a binary image is a discrete representation of two-dimensional space, we use Bresenham’s line algorithm [36] to search grids that can approximately represent a straight line in binary-image. The envelopes of obstacles are shown in Figure 14a. There are some gaps between the envelope and the boundary of the obstacle. If these gaps are not filled, the skeleton of these gaps will also be extracted in the subsequent skeleton extraction process, which reduces the efficiency of skeleton extraction for binary-image. Therefore, we use a hole-filling algorithm based on contours to fill these gaps [37]. Figure 14b shows the results of gap filling, which is also the end result of convex hull transform. Then we can obtain a smooth skeleton of navigable regions, as shown in Figure 14c.

**Figure 15:**
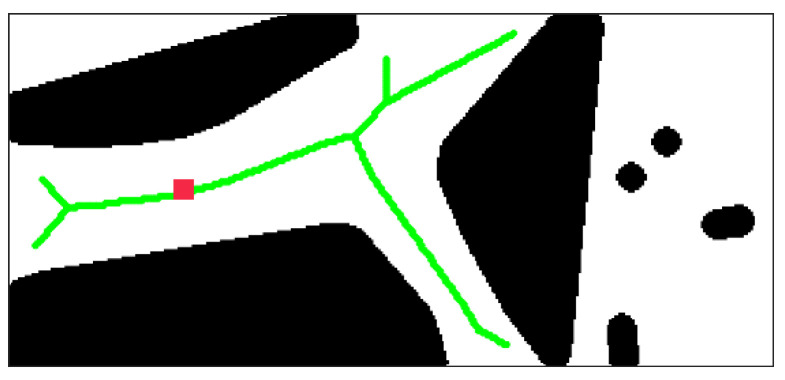
The searching result of the desired skeleton.

There may be several unconnected navigable regions in OGM, among which only the skeleton of the navigable region where the UGV is currently located is the desired skeleton. In order to reduce the computational load, it is necessary to find out the skeleton corresponding to the current position of the UGV. The basic idea of this process is transforming the skeleton search problem into a data classification problem by using the k-nearest neighbor (kNN) algorithm [38]. The position data of all skeleton points of each skeleton was regarded as a sample set, and the position of the UGV was regarded as the data to be classified. The correlation between the data to be classified and the sample is judged by calculating the distance between them. Euclidean distance was used to measure the distance between the test data and all the training samples, which is defined as:

(20) $$D\left(x,y\right)={\left(\overset{m}{\underset{i=1}{\sum }}{\left|x_{i}-y_{i}\right|}^{2}\right)}^{\frac{1}{2}}\;,$$

where D(x,y) represents distance between point *x* and *y*, and m=2 in two-dimensional space. The searching result of the desired skeleton is shown in Figure 15. The red square indicates the position of the UGV in the diagram.

#### 3.3.3. Optimization of Skeleton Shape

The Zhang–Suen algorithm is a thinning method based on the grassfire transform. It can be described as “setting fire” to the borders of an image region to yield descriptors such as the region’s skeleton or medial axis. However, any point can only travel in a maximum of 8 directions in a discrete two-dimensional space, so it is hard to simulate arbitrary burning direction. Therefore, when the skeleton is extracted from the region with irregular shape, it is likely to generate a skeleton branch that is biased towards the obstacle. In this paper, a skeleton shape optimization method based on gradient descent is proposed to solve the above problem.

First of all, the skeleton is divided into two parts: trunk and branch. The segment connecting two fork points in the skeleton is defined as the trunk. The segment connecting a fork point and an endpoint is defined as the branch. It can be seen that the precondition of skeleton decomposition is to find the fork points and endpoints accurately, and the corresponding discriminant conditions are as follows:

- The definition of the foreground pixel $P_{1}$ and its eight neighborhoods in Section 3.3.1 is followed.
- $M\left(P_{1}\right)=P_{2}+P_{3}+P_{4}+P_{5}+P_{6}+P_{7}+P_{8}+P_{9}$;
- Iterate through all the pixels in the foreground;
- Mark the pixel with $M\left(P_{1}\right)=1$ as the endpoint;
- Mark the pixel with $M\left(P_{1}\right)=3$ or $M\left(P_{1}\right)=4$ as fork point.

**Figure 16:**
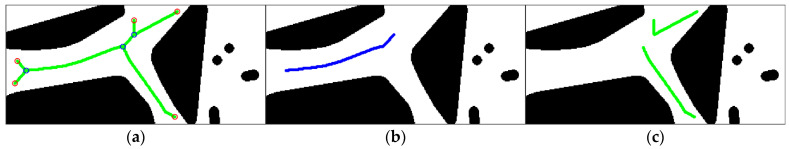
The process of skeleton decomposition. (**a**) Fork points and endpoints of the skeleton; (**b**) the skeleton trunk; (**c**) the skeleton branch ahead of the UGV.

The searching result of fork points and endpoints is shown in Figure 16a, and blue and red circles indicate fork points and endpoints respectively. For efficiency, we only deal with the skeleton branch ahead of the UGV. The result of skeleton decomposition is shown in Figure 16b,c.

**Figure 17:**
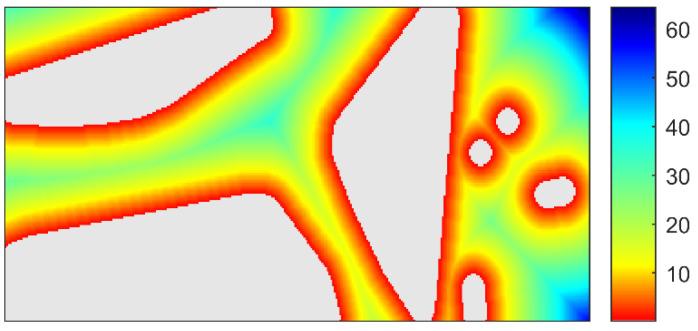
An example of the occupied grid map (OGM) distance field.

In order to apply the gradient descent algorithm, we constructed a distance field of the OGM utilizing distance transform method [39]. The distance transformation of the OGM is to calculate the minimum distance from each non-occupied grid to the grid of obstacle boundary. When there are m isolated obstacles in the OGM, the distance field can be expressed as follows:

(21) $$F_{d}\left(k\right)=\underset{}{\min}\left(d\left(k,S\left[i\right]\right)\right),\;i=1,2,\cdots ,m,$$

where $F_{d}\left(k\right)$ is the value of the distance field; d(,) is Euclidean distance calculation function; k is an arbitrary non-occupied grid; S is collection of obstacle boundary grids. An example of constructed distance field is shown in Figure 17.

Gradient descent is a first-order iterative optimization algorithm for finding a local minimum of a differentiable function. The idea is to take repeated steps in the opposite direction of the gradient (or approximate gradient) of the function at the current point, because this is the direction of steepest descent. Through each iteration, the function to be optimized is gradually reduced, so as to solve the minimum value of the function. The iterative translation process of skeleton points based on gradient descent can be described by the formula:

(22) $$x_{i,k+1}=x_{i,k}-a_{k}g\left(x_{i,k}\right),$$

where $x_{i,k}$ is the position of the *i-th* skeleton point obtained after the *k-th* iteration; $g\left(x_{i,k}\right)$ is the gradient magnitude of the *i-th* skeleton point at position $x_{i,k}$; $a_{k}$ is the iteration step length.

Because the distance field of OGM is a discrete two-dimensional plane, we utilize horizontal and vertical convolution to calculate the horizontal and vertical gradient values of each position respectively, and then the gradient magnitude can be obtained by using Equation (23).

(23) $$g\left(x_{i,k}\right)=\sqrt{{\left|S_{x}\right|}^{2}+{\left|S_{y}\right|}^{2}},$$

where horizontal gradient value $S_{x}$ and vertical gradient value $S_{y}$ are calculated by Equations (24) and (25) respectively.

(24) $$S_{x}=f\left(i+1,j+1\right)+2f\left(i+1,j\right)+f\left(i+1,j-1\right)-\;f\left(i-1,j+1\right)-2f\left(i-1,j\right)-f\left(i-1,j-1\right),$$

(25) $$S_{y}=f\left(i+1,j+1\right)+2f\left(i,j+1\right)+f\left(i-1,j+1\right)-\;f\left(i-1,j-1\right)-2f\left(i,j-1\right)-f\left(i+1,j-1\right),$$

**Figure 18:**
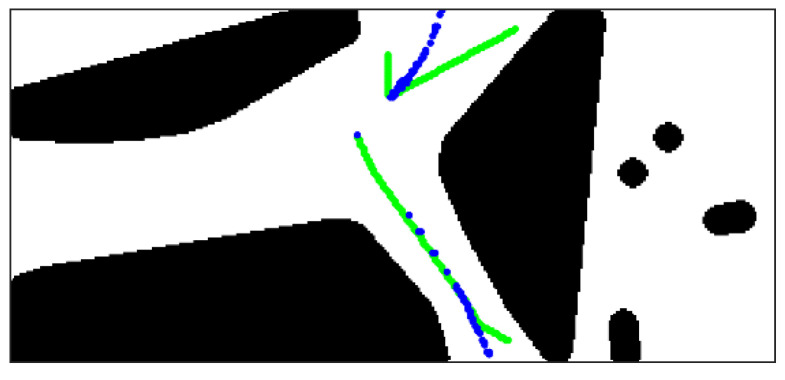
The result of skeleton shape optimization.

The termination condition of iteration of the gradient descent algorithm is defined as: Let $g\left(x_{i,k}\right)$ and $g\left(x_{i,k-1}\right)$ be the gradient magnitude of skeleton point $x_{i}$ for two consecutive iterations, and ε be the difference threshold of the gradient magnitude, when meet condition $\left|g\left(x_{i,k}\right)-g\left(x_{i,k-1}\right)\right|<\varepsilon$, the iteration ends. As shown in Figure 18, the green points are the original skeleton branches, and the blue points are the new distribution of the skeleton points after the translation.

#### 3.3.4. Candidate Guidance Point Generation

**Figure 19:**
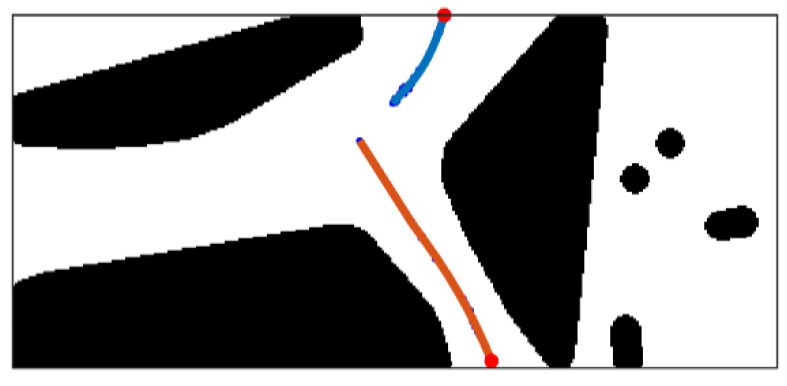
The result of skeleton shape optimization.

After the translation by the gradient descent method, the topological connection between the skeleton points is destroyed, so it is necessary to construct a new skeleton bend based on the current spatial distribution of the skeleton points. In this paper, we use cubic polynomial to fit the skeleton points. As can be seen from Figure 19, the skeleton branches obtained by fitting are close to the midline position of the navigable area.

Taking the end point of the new skeleton branch (red points in Figure 19) as the expected target position of UGV, and taking the tangent direction of the curve at this position as the expected heading, multiple candidate guidance points containing position and heading information can be obtained. The candidate guidance point can be defined as follows:

(26) $$x_{i,t}=x_{i,n},$$

(27) $$y_{i,t}=y_{i,n},$$

(28) $$\theta =arctan\left(\frac{\partial f_{i}}{\partial x}\left(x_{i,n},\;y_{i,n}\right)\right),$$

where $\left(x_{i,t},y_{i,t}\right)$ and θ represent position and heading of candidate guidance point, $\left(x_{i,n},y_{i,n}\right)$ is the end point of the skeleton branch, $f_{i}\left(x,y\right)$ is the curve function of *i-th* skeleton branch.

## 4. Human-Machine Cooperation-Based UGV Teleoperation

When the candidate guidance point is generated by the environment perception system, the operator needs to select the appropriate guidance point through the teleoperation system as the tracking target of UGV autonomous maneuvering. This human–machine cooperative control mode based on guidance information interaction transforms the operator’s high-frequency driving control behavior into low-frequency decision behavior, which further decouples the operator and UGV, thus reducing the sensitivity of the control loop to delay. This section will give a detailed introduction to the human–machine interaction process in collaborative control.

### 4.1. Human–Machine Interaction Mechanism Design

**Figure 20:**
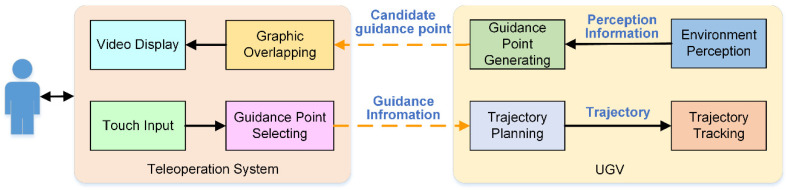
Human–machine interaction for cooperative control.

Human–machine cooperative control based on guidance information interaction realizes a hierarchical cooperation control through the information interaction among operator, teleoperation system and the UGV. In this process, first of all, candidate guidance points need to be superimposed in the UGV feedback video. In order to make the candidate guidance points overlay the dynamic image always on a fixed position in the scene, first of all, we need to convert the relative position of the guidance point in the vehicle coordinate system to the absolute position in the global coordinate system. Then, based on the transformation relationship between the global coordinate system and the image coordinate system, the candidate guidance points are superimposed to each frame of the video. In addition, for the intuitive and efficient human-machine interaction, this paper chooses the touch screen as the human interface device of the teleoperation system. The basic process of human–machine interaction is shown in Figure 20.

The interaction mechanism of human-machine system is as follows:

1. The autonomous control system generates candidate guidance points periodically and sends them to the teleoperation system.
2. The teleoperation system overlays the candidate guidance points with the video images and displays them.
3. The operator selects a candidate point as the guidance point of the UGV according to the control intention.
4. The teleoperation system sends the selected guidance point back to the UGV, and the autonomous control system takes the guidance point as the input to generate the desired trajectory line based on the kinematics constraints of the UGV.
5. During trajectory tracking, if the operator selects a new guidance point, the UGV will immediately generate trajectory based on the new guidance point.
6. When the UGV is close to the current guidance point, if the operator has not yet provided a new guidance point, the autonomous control system will select a candidate guidance point as the tracking target point by itself according to the principle of minimum trajectory curvature.
7. If no new guidance point is available, the UGV will stop automatically.

### 4.2. Trajectory Generation Based On Kinematic Constraints

**Figure 21:**
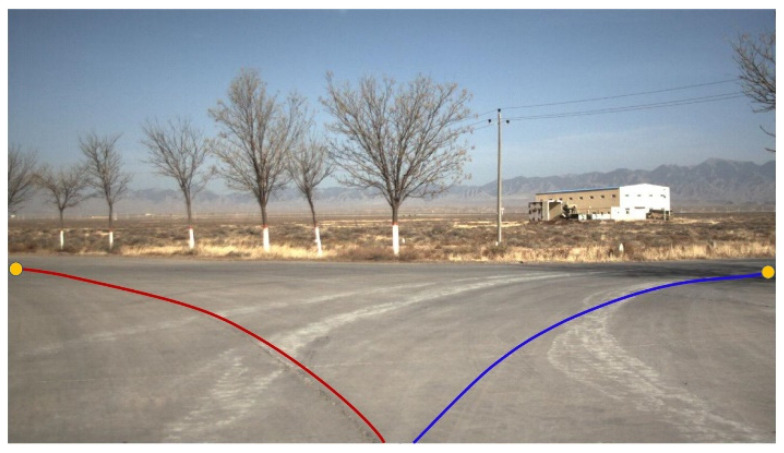
Superimposed candidate trajectories on the video image.

When the UGV is running at a high speed, the scene in the video changes rapidly, leading to the position of the guide point on the screen moving rapidly, which makes the operator fail to select the guide point. In addition, it is not helpful for the operator to know the future driving trend of the UGV only by marking several candidate guidance points on the video image. Therefore, a more effective way to display guidance information is to superimpose several candidate trajectories on the video image, as shown in Figure 21.

The advantage of using trajectory superposition is that the pixel region corresponding to the entire trajectory can respond to the operator’s click operation, thus enlarging the response region of touch operation from a small pixel region to a banded pixel region. The operator does not have to carefully click on a location on the screen, which greatly improves the success rate of the operation. Another benefit is that, by observing the predicted driving trajectory, the operator can intuitively know the locations in the scene where the UGV will probably pass in the next period of time, which is conducive to the operator to make forward-looking and safe decisions.

In order to make the superimposed trajectory line conform to the actual driving state of the UGV, a candidate trajectory line is generated based on the motion differential constraint of the UGV.

(29) $$\dot{x}=v\times \cos\left(\theta \right),$$

(30) $$\dot{y}=v\times \sin\left(\theta \right),$$

(31) $$\dot{\theta }=v\times \frac{\tan\left(\delta \right)}{L},$$

where (x,y) is the current position of the UGV, θ is the heading angle, v is the speed, L is the wheelbase, and δ is the front wheel angle. According to the reference [40], the curve satisfying the above differential equations of motion can be described by polynomial parametric equation of degree n (n≥3). Therefore, the position of the UGV can be expressed as follows:

(32) x=x(u), u∈[0,1],

(33) y=y(u), u∈[0,1],

where x(u) and y(u) represent polynomial parametric equations of x and y coordinates, u is dimensionless parameter, (x(0),y(0)) and (x(1),y(1)) represent the coordinates of the starting and end positions. The parametric equation transforms the problem of solving the trajectory curve into the problem of solving the coefficient of the parametric equation. According to the current posture $\left(x_{S},y_{S},\delta_{S},\;\theta_{S},\right)$ and the target posture $\left(x_{E},y_{E},\delta_{E},\;\theta_{E},\right)$, the following boundary conditions can be obtained:

(34) $$x\left(0\right)=x_{S},\;x\left(1\right)=x_{E},$$

(35) $$y\left(0\right)=y_{S},\;y\left(1\right)=y_{E},$$

(36) $$\delta \left(0\right)=\delta_{S},\delta \left(1\right)=\delta_{E},$$

(37) $$\frac{\left[\begin{array}{c} \dot{x}\left(0\right)\\ \dot{y}\left(0\right) \end{array}\right]}{\sqrt{x^{2}\left(0\right)+y^{2}\left(0\right)}}=\left[\begin{array}{c} \cos\theta_{S}\\ \sin\theta_{S} \end{array}\right],$$

(38) $$\frac{\left[\begin{array}{c} \dot{x}\left(1\right)\\ \dot{y}\left(1\right) \end{array}\right]}{\sqrt{x^{2}\left(1\right)+y^{2}\left(1\right)}}=\left[\begin{array}{c} \cos\theta_{E}\\ \sin\theta_{E} \end{array}\right].$$

The above boundary conditions can determine the unique solution of the cubic polynomial parametric equation. In order to make the trajectory curve have more degrees of freedom and reduce the impact strength on the curve, the polynomial parametric equations of degree 5 was selected to model the trajectory curve, which are given as follows:

(39) $$x\left(u\right)=x_{0}+x_{1}u+x_{2}u^{2}+x_{3}u^{3}+x_{4}u^{4}+x_{5}u^{5},$$

(40) $$y\left(u\right)=y_{0}+y_{1}u+y_{2}u^{2}+y_{3}u^{3}+y_{4}u^{4}+y_{5}u^{5}.$$

To solve the coefficients of the indefinite equations, we add additional adjustable parameters $\left(\epsilon_{1},\;\epsilon_{2},\;\epsilon_{3},\epsilon_{4}\right)$ so as to convert Equations (39) and (40) to explicit equations of coefficients versus adjustable parameters [40], and the optimal solution can be determined.

**Figure 22:**
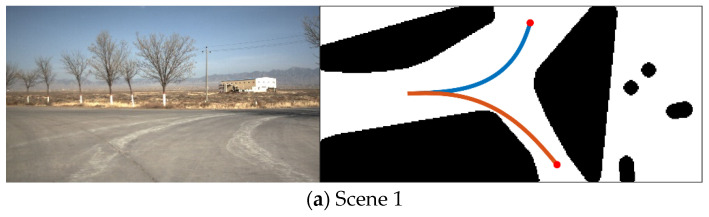

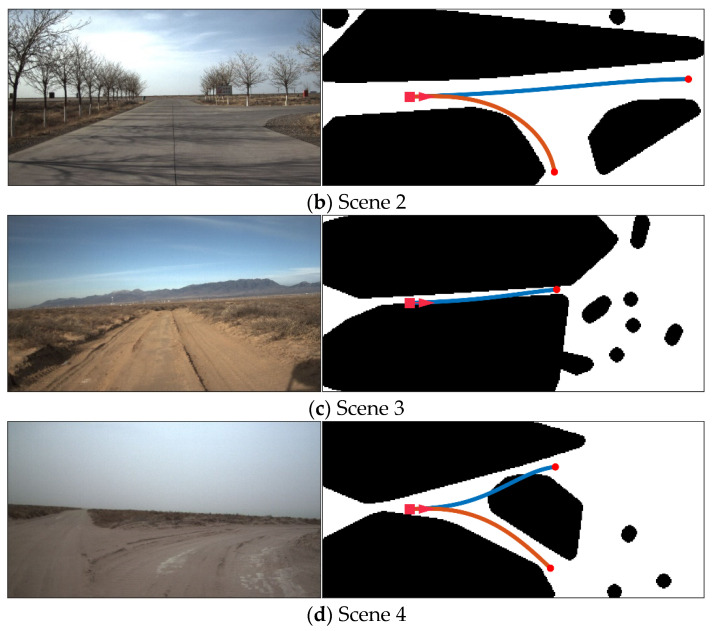
The trajectory curves satisfying the differential constraint of motion in different scenes.

In order to keep the length of the curve short while the change rate of the maximum curvature is as small as possible, the objective function of the optimization solution is defined as:

(41) $$\underset{}{\min}\left(\underset{}{\max}\left(\left|\dot{\kappa }\right|\right)\times k+s\right),$$

where κ denotes the curvature of the curve, s is the curve length, and k is the weighting coefficient. Figure 22 shows the trajectory curves calculated according to the same starting position and heading and different ending position and heading.

## 5. Experiments

In this section, the performance of the suggested human-machine cooperative control method is investigated under various in unstructured environments.

### 5.1. Test System

The test system is composed of wheeled UGV, teleoperation system and wireless communication system.

#### 5.1.1. Wheeled Unmanned Ground Vehicle (UGV)

**Figure 23:**
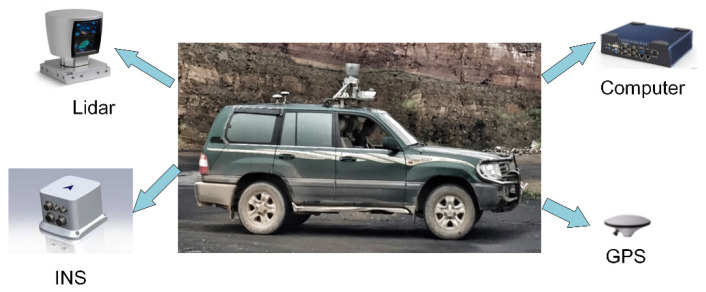
Components of the navigation control system.

The UGV used in the experiment was modified from a wheel off-road vehicle with a length of 5.1 m and a width of 1.97 m. The navigation control system of the UGV consisted of environment perception sensors and an on-board computer, as shown in Figure 23. Environment perception sensors included a Velodyne HDL-64E lidar, a real-time kinematic (RTK) GPS system, and a fiber-optic inertial navigation system (INS). The vertical and horizontal views of the lidar were 26.8° and 360°. We could obtain 1.3 million points per second with a range accuracy of +/−2 cm. The INS had the horizontal position accuracy of 0.02 m~0.05 m and the altitude position accuracy of 0.02 m~0.08 m, and the horizontal attitude accuracy was within 0.01°. The on-board computer is a ruggedized machine (Intel Core i7-8550U 1.8 GHz, Intel HD Graphics 620, 512G Solid State Drive GeForce 8800 Ultra video, and 4 GB memory) with the 64-bit Linux operating system (Ubuntu 18.04). It implemented intelligent decision making and control, and ensured real-time operation of software such as local path planning and tracking control.

#### 5.1.2. Teleoperation System

**Figure 24:**
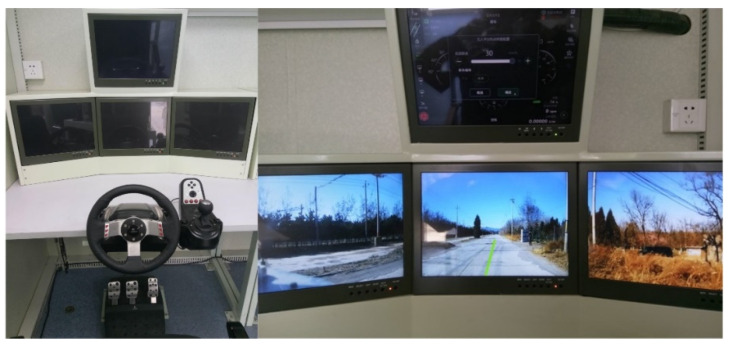
Teleoperation system.

The teleoperation system was housed in a mobile control shelter which provided sufficient space, power, and environmental control for the operator, as shown in Figure 24. The operator received video and state feedback data on display terminal and issued driving commands through UGV controller and touch screens. The teleoperation computer had the same hardware configuration as the navigation control computer. A multi-screen display system was applied to display the wide-field-of-view video and graphical user interface. The Logitech G27 driving device was used for manual driving.

#### 5.1.3. Wireless Communication System

The wireless communication system between the teleoperation system and the UGV was carried by a mobile ad hoc network (MANET) [41]. The MANET was a collection of wireless mobile nodes dynamically forming a temporary network without the use of any existing network infrastructure or centralized administration. The frequency band of the communication system was 560~678 MHz, which could provide the data bandwidth of 8 Mbps, and the link transmission delay was less than 20 ms. In flat terrain environment, the maximum communication distance could reach 10 km. For ease of implementation, teleoperation commands and feedback information travelled over the same network.

### 5.2. Experimental Design

**Figure 25:**
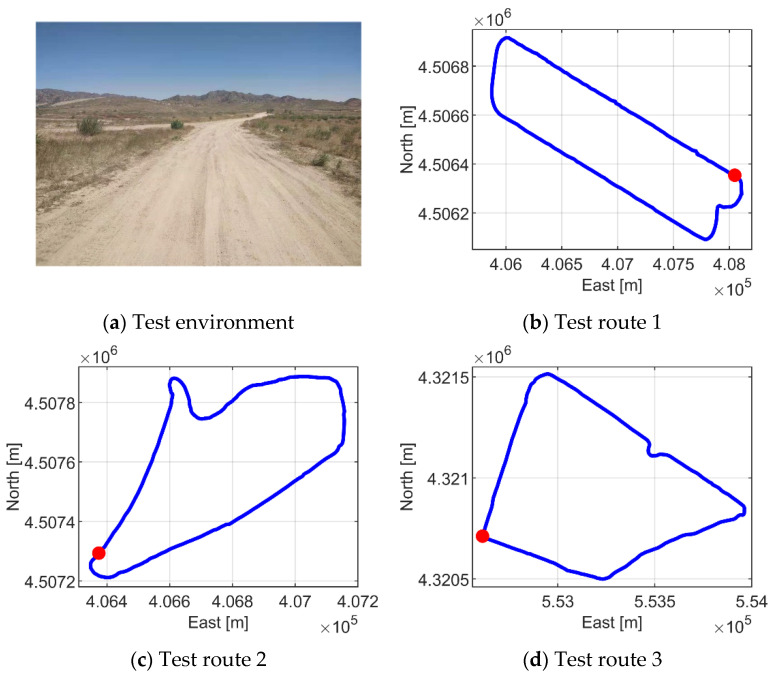
Test environment and route.

We conducted a human-in-the-loop field experiment on a semi-desert region in northwest China. The experiment was carried out in the daytime without rain or snow. Three closed experimental routes were designed in an unstructured environment, each of which was about 4.96 km, 2.4 km and 3.4 km in length, including typical environmental elements such as straight, curve, pit and mound, and the starting point and the end point were the same, as shown in Figure 25. There were signs at the fork and endpoint to inform participants where to make a turn and when to stop. In order to ensure the safety of the experiment, the maximum speed of the UGV was limited to 55 km/h, 55 km/h and 40 km/h, respectively, depending on the road conditions of the three routes.

Four operators were selected as the subjects to carry out experiments in three test routes. Among the operators, there were three males and one female, aged between 23 and 30, and all of them had UGV remote control driving experience. They were asked to driving the UGV inside the road as far as possible. Before the experiment, the operator was allowed to practice freely in the experimental site for a while to fully adapt to the test system. It also allowed the operator to preview the full course of the test route using a digital map. The operator had no direct eye contact with the UGV in the field. For each experiment route, the operator used the remote control mode (manual driving) and the cooperative control mode to control the UGV running counterclockwise along the test route respectively, and recorded the motion information of each lap. Subjects drew lots to determine the experimental order. While one subject was experimenting, the other subjects were not allowed to look. A 5-min break was given between the modes. If there was a serious accident such as the UGV falling into a pit or crashing into a mound, the experiment would be judged a failure. It would be judged according to its actual mileage to evaluate the completion rate of this task.

### 5.3. Experimental Result

We evaluated the performance of the proposed cooperative control method from two aspects: maneuvering task performance and handling stability.

#### 5.3.1. Maneuvering Task Performance

The task completion rate and average speed are usually used to evaluate the maneuvering task performance of a UGV [28,42]. The task completion rate reflects the extent to which the UGV completes a particular task under the operator’s control, which can be defined as follows:

(42) $$Task\;Completion\;Rate=\frac{Length\;of\;Driven\;Route}{Task\;Route\;Length}\times 100\%.$$

During the experiment, four operators used two teleoperation modes to complete all three routes safely, and the completion rate of the task reached 100%.

**Figure 26:**
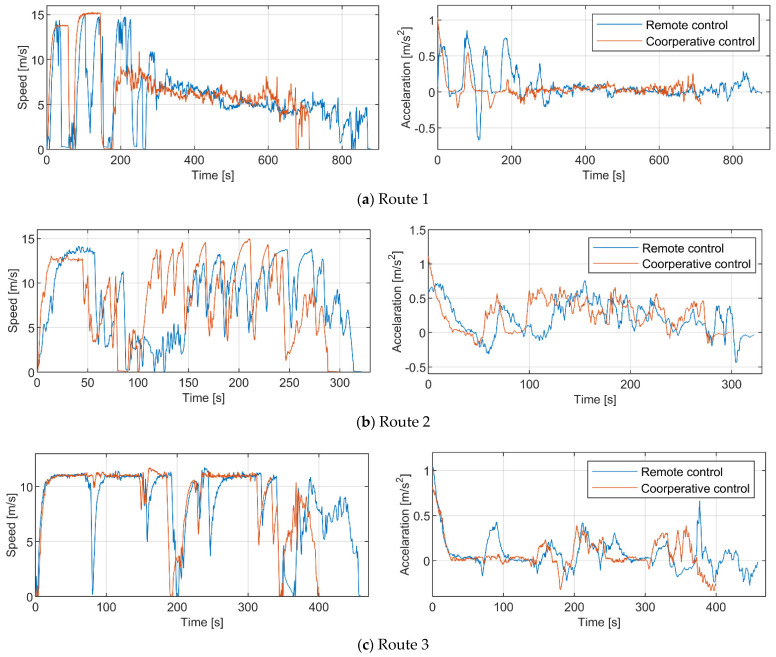
Driving speed and acceleration variation in three test routes based on remote control mode and human-machine cooperative control mode from one of the subjects.

The average speed represents the completion efficiency of the maneuver task. The higher the average speed, the greater the maneuverability of the UGV in the task environment, and the shorter the time to complete the task. Figure 26 shows the driving speed and acceleration in different test routes based on remote control mode and human–machine cooperative control mode from one of the subjects. As can be seen from the chart, both modes can achieve a similar maximum speed, but the speed control stability of the former is better than that of the latter.

Table 1 shows the average driving speed of each subject in remote control and human–machine cooperative control mode in three routes. By comparing the mean values of average speed between these two modes in each test route, we can see that human–machine cooperative control mode has advantages for UGV teleoperation in an unstructured environment.

#### 5.3.2. Handling Stability

In the field of vehicle engineering, yaw rate and sideslip angle are important indicators to measure the handling stability of a vehicle [43]. The yaw rate γ, heading φ, velocity components $v_{x}$ and $v_{y}$ in global coordinates can be measured by the onboard INS. Based on the Ackerman bicycle model, the sideslip angle β is calculated by Equation (43) [44]:

(43) β=θ−φ,

where θ is the direction angle of velocity, which is defined as:

(44) $$\theta =\tan^{-1}\frac{v_{y}}{v_{x}},$$

In this experiment, we used the mean absolute deviation (MAD) of yaw rate and sideslip angle to evaluate the handling stability of UGV. The MAD of a dataset is the average distance between each data point and the mean. It gives us an idea about the variability in a dataset. The MAD of a dataset $\left\{x_{1},\;x_{2},\cdots ,x_{n}\right\}$ is defined as:

(45) $$D=\frac{1}{n}\overset{n}{\underset{i=1}{\sum }}\left|x_{i}-m\left(X\right)\right|,$$

where m(X) is the mean of dataset X. Table 2 and Table 3 show the MAD of yaw rate and sideslip angle respectively.

### 5.4. Experiment Analysis

The purpose of the experiment was to verify the usability of the proposed human–machine cooperative control method in multiple unstructured environments. The four operators all completed the maneuvering tasks of the three test routes safely by using the cooperative control method.

Through the comprehensive analysis of the speed data and task routes, it can be seen that both modes can achieve a similar maximum speed on the straight section of each route. But due to the delay in the control loop, the handling stability of the remote control mode decreases when the UGV is driving at high speed, which makes it difficult for the operator to maintain the high-speed driving state for a long time. To ensure driving safety, the operator will take the initiative to reduce the driving speed even under good road conditions. Moreover, due to the limited visual field of the video image, the operator will also slow down in the remote control mode during sharp turns or continuous curves to ensure the UGV can pass safely. For task routes with more curves, this will lead to longer task completion time. It can also be seen from Figure 26 that the fluctuation of the acceleration curve in the remote control mode is significantly greater than that in the cooperative control mode.

In the human–machine cooperative control mode, the control command generated by the teleoperation system is the guidance point that is spatially ahead of the current UGV position. The desired path is generated by the navigation control system according to the guidance point, then the UGV can drive along the path at the optimal speed and course under precise control. Compared with the operator’s direct control, the control accuracy and frequency of the autonomous control system to the UGV chassis are higher, so it can continuously maintain a higher speed, and pass the curve section more smoothly.

## 6. Conclusions

This paper focuses on guidance point generation-based cooperative UGV teleoperation in an unstructured environment. The key novelty of this method is that the guidance points used for navigation can be generated with only the local perception information of UGV. In order to extract smooth skeletons of navigable regions, the OGM was generated utilizing a probabilistic grid state description method, and converted into binary images to construct the convex hull of the obstacle area. An improved thinning algorithm is designed to extract single-pixel skeleton, and find out the target skeleton related to the position of UGV utilizing the kNN algorithm. By using the decreasing gradient algorithm the target skeleton is modified in order to obtain the appropriate candidate guidance points. For visually presenting the driving trend of the UGV and convenient touch screen operation, we transformed guidance point selection into trajectory selection by generating the predicted trajectory correlative to candidate guidance points based on the differential equation of motion. Experimental results show that the cooperative control mode is suitable for UGV teleoperation in off-road environments.

The advantage of the proposed method is that the operator only needs to select the guiding point consistent with its control intention as the target point of UGV trajectory tracking to realize the teleoperation. Compared with the traditional remote-control method, this human–machine cooperative control mode transforms the operator’s high-frequency control behavior (e.g., steering, accelerating and braking actions) into low-frequency decision-making behavior, which further decouples the operator from the UGV, thus reducing the workload of the operator and the sensitivity of the control loop to delay.

In the remote control mode, all motion control commands are input by the operator. This means that the operator needs to train for a while to adapt to the handling characteristics and delay effects of the UGV. When the controlled vehicle changes, the operator also needs to be retrained. In comparison, the human–machine cooperative control method is weakly correlative with the vehicle type. The operator is not concerned with the motion control of the vehicle. By using this method, the operator can obtain acceptable teleoperation ability after a few elements of training. Therefore, we believe that this method should have broad application prospects.

The limitation of the method proposed in this paper lies in the fact that the grid occupied state is not considered when generating the trajectory line, and there is a risk of collision with obstacles in extreme cases. Therefore, the trajectory cluster-generating method will be studied in following research. The safe trajectory will be selected from the trajectory cluster for UGV tracking.

Since the onboard camera of the UGV does not have night vision ability, images taken in the night environment cannot be used for the operator to make decisions, so current research is only based on the daytime environment. In following research work, we will choose a camera with low illumination imaging ability to carry out the research on human–machine cooperative teleoperation in the night environment.

## Figures and Tables

**Table 1:** Average driving speed of each subject in remote control and human–machine cooperative control mode in three routes.

Subject	Average Speed of Route 1 [m/s]		Average Speed of Route 2 [m/s]		Average Speed of Route 3 [m/s]	
	R. C.	H. C. C.	R. C.	H. C. C.	R. C.	H. C. C.
1	5.70	6.96	7.98	8.34	8.66	9.17
2	6.38	5.94	5.67	6.83	7.02	7.89
3	5.93	6.33	7.58	6.17	9.61	10.36
4	5.24	5.78	8.86	9.84	7.37	7.61
Mean Value	5.81	6.25	7.52	7.80	8.17	8.76

R. C., Remote Control; H. M. C., Human–machine Cooperative Control.

**Table 2:** Mean absolute deviation (MAD) of yaw rate of each subject in remote control and human–machine cooperative control mode in three routes.

Subject	MAD of Yaw Rate in Route 1 [°/s]		MAD of Yaw Rate in Route 2 [°/s]		MAD of Yaw Rate in Route 3 [°/s]	
	R. C.	H. C. C.	R. C.	H. C. C.	R. C.	H. C. C.
1	0.97	0.79	2.68	2.78	1.52	0.96
2	1.10	0.90	2.30	3.48	1.84	1.21
3	1.13	0.69	2.38	2.59	1.62	0.91
4	0.95	0.67	3.61	2.92	1.38	1.00
Mean Value	1.04	0.76	2.74	2.94	1.59	1.02

**Table 3:** MAD of sideslip angle of each subject in remote control and human-machine cooperative control mode in three routes.

Subject	MAD of Sideslip Angle in Route 1 [°]		MAD of Sideslip Angle in Route 2 [°]		MAD of Sideslip Angle in Route 3 [°]	
	R. C.	H. C. C.	R. C.	H. C. C.	R. C.	H. C. C.
1	0.25	0.16	0.45	0.37	0.33	0.21
2	0.24	0.21	0.51	0.40	0.41	0.19
3	0.20	0.14	0.54	0.36	0.29	0.26
4	0.18	0.15	0.33	0.43	0.37	0.24
Mean Value	0.22	0.17	0.46	0.42	0.35	0.23

## Data Availability

Not applicable.

## References

[1] Chen J.Y., Haas E.C., Barnes M.J. (2007). Human performance issues and user interface design for teleoperated robots. IEEE Trans. Syst. Man Cyber. Part C Appl. Rev..

[2] Luck J.P., McDermott P.L., Allender L., Russell D.C. An investigation of real world control of robotic assets under communication latency. Proceedings of the 1st ACM SIGCHI/SIGART Conference on Human-Robot Interaction.

[3] Storms J., Tilbury D. Equating user performance among communication latency distributions and simulation fidelities for a teleoperated mobile robot. Proceedings of the IEEE International Conference on Robotics and Automation (ICRA).

[4] Ferland F., Pomerleau F., Le Dinh C.T., Michaud F. Egocentric and exocentric teleoperation interface using real-time, 3d video projection. Proceedings of the 4th ACM/IEEE International Conference on Human-Robot Interaction (HRI).

[5] Badue C., Guidolini R., Carneiro R.V., Azevedo P., Cardoso V.B., Forechi A., Jesus L., Berriel R., Paixão T.M., Mutz F. (2020). Self-driving cars: A survey. Expert. Syst. Appl..

[6] Yurtsever E., Lambert J., Carballo A., Takeda K. (2020). A survey of autonomous driving: Common practices and emerging technologies. IEEE Access.

[7] Cosenzo K.A., Barnes M.J., Martin J., Allender L., Savage-Knepshield P., Lockett J. (2018). Who needs an operator when the robot is autonomous? The challenges and advantages of robots as team members. Designing Soldier Systems: Current Issues in Human Factors.

[8] Kelly A., Chan N., Herman H., Warner R., Desai J.P., Dudek G., Khatib O., Kumar V. (2013). Experimental validation of operator aids for high speed vehicle teleoperation. Experimental Robotics.

[9] Macharet D.G., Florencio D. A collaborative control system for telepresence robots. Proceedings of the IEEE/RSJ International Conference on Intelligent Robots and Systems (IROS).

[10] Anderson S.J., Walker J.M., Iagnemma K. (2014). Experimental performance analysis of a homotopy-based shared autonomy framework. IEEE Trans. Humman-Mach. Syst..

[11] Erlien S.M., Funke J., Gerdes J.C. Incorporating non-linear tire dynamics into a convex approach to shared steering control. Proceedings of the IEEE American Control Conference (ACC).

[12] Shia V., Gao Y., Vasudevan R., Campbell K.D., Lin T., Borrelli F., Bajcsy R. (2014). Semiautonomous vehicular control using driver modeling. IEEE Trans. Intell. Transp. Syst..

[13] Bicker R., Ow S.M. Shared control in bilateral telerobotic systems. Proceedings of the SPIE—The International Society for Optical Engineering.

[14] Brandt T., Sattel T., Bohm M. Combining haptic human-machine interaction with predictive path planning for lane-keeping and collision avoidance systems. Proceedings of the IEEE Intelligent Vehicles Symposium.

[15] Mulder M., Abbink D.A., Boer E.R. (2012). Sharing control with haptics: Seamless driver support from manual to automatic control. Hum. Factors.

[16] Flemisch F., Heesen M., Hesse T., Kelsch J., Schieben A., Beller J. (2012). Towards a dynamic balance between humans and automation: Authority, ability, responsibility and control in shared and cooperative control situations. Cogn. Technol. Work.

[17] Gray A., Ali M., Gao Y., Hedrick J., Borrelli F. Semi-autonomous vehicle control for road departure and obstacle avoidance. Proceedings of the IFAC Symposium on Control in Transportation Systems.

[18] Petermeijer S.M., Abbink D.A., de Winter J.C. (2015). Should drivers be operating within an automation-free bandwidth? Evaluating haptic steering support systems with different levels of authority. Hum. Factors.

[19] Forsyth B.A., Maclean K.E. (2006). Predictive haptic guidance: Intelligent user assistance for the control of dynamic tasks. IEEE Trans. Visual. Comput. Graph..

[20] Mulder M., Abbink D.A., Boer E.R. The effect of haptic guidance on curve negotiation behavior of young, experienced drivers. Proceedings of the IEEE International Conference on Systems, Man and Cybernetics.

[21] Abbink D.A., Cleij D., Mulder M., Van Paassen M.M. The importance of including knowledge of neuromuscular behaviour in haptic shared control. Proceedings of the IEEE International Conference on Systems, Man, and Cybernetics.

[22] Mars F., Deroo M., Hoc J.M. (2014). Analysis of human-machine cooperation when driving with different degrees of haptic shared control. IEEE Trans. Haptics.

[23] Boehm P., Ghasemi A.H., O’Modhrain S., Jayakumar P., Gillespie R.B. (2016). Architectures for shared control of vehicle steering. IFAC-PapersOnLine.

[24] Witus G., Hunt S., Janicki P. (2011). Methods for UGV teleoperation with high latency communications. Unmanned Systems Technology XIII.

[25] Silver D., Sofman B., Vandapel N., Bagnell J.A., Stentz A. Experimental analysis of overhead data processing to support long range navigation. Proceedings of the IEEE/RSJ International Conference on Intelligent Robots and Systems.

[26] Zych N., Silver D., Stager D., Green C., Pilarski T., Fischer J., Kuntz N., Anderson D., Costa A., Gannon J. (2013). Achieving integrated convoys: Cargo unmanned ground vehicle development and experimentation. Unmanned Systems Technology XV.

[27] Liu D. (2011). Research on Human-machine Intelligent Integration Based Path Planning for Mobile Robots. Master’s Thesis.

[28] Suzuki T., Amano Y., Hashizume T., Kubo N. (2013). Vehicle teleoperation using 3D maps and GPS time synchronization. IEEE Comput. Graph. Appl..

[29] Kaufman E., Lee T., Ai Z., Moskowitz I.S. Bayesian occupancy grid mapping via an exact inverse sensor model. Proceedings of the IEEE American Control Conference (ACC).

[30] Dougherty E. (2018). Mathematical Morphology in Image Processing.

[31] Xie W., Thompson R.P., Perucchio R. (2003). A topology-preserving parallel 3D thinning algorithm for extracting the curve skeleton. Pattern Recognit..

[32] Ding Y., Liu W.Y., Zheng Y.H. (2005). Hierarchical connected skeletonization algorithm based on distance transform. J. Infrared Millim. Waves.

[33] Ogniewicz R.L. (1995). Hierarchic Voronoi skeletons. Pattern Recognit..

[34] Zhang T.Y., Suen C.Y. (1984). A fast parallel algorithm for thinning digital patterns. Commun. ACM.

[35] Graham R.L. (1972). An efficient algorithm for determining the convex hull of a finite planar set. Info. Pro. Lett..

[36] Bresenham J.E. (1965). Algorithms for computer control of a digital plotter. IBM Syst. J..

[37] Zhang D.C., Zhou C.G., Zhou Q., Chi S.Z., Wang S.J. (2011). Hole-filling algorithm based on contour. J. Jilin Uni..

[38] Rajaguru H., Prabhakar S.K. (2017). KNN Classifier and K-Means Clustering for Robust Classification of Epilepsy from EEG Signals.

[39] Saito T., Toriwaki J.I. (1994). New algorithms for euclidean distance transformation of an n-dimensional digitized picture with applications. Pattern Recognit..

[40] Broggi A., Bertozzi M., Fasciolia A., Guarino C., Lo Bianco C.G., Piazzi A. (1999). The ARGO autonomous vehicles vision and control systems. Int. J. Intell. Cont. Syst..

[41] Vegda H., Modi N. (2018). Review paper on mobile ad-hoc networks. Int. J. Comput. Appl..

[42] Zheng Y., Brudnak M.J., Jayakumar P., Stein J.L., Ersal T. (2016). An experimental evaluation of a model-free predictor framework in teleoperated vehicles. IFAC-PapersOnLine.

[43] Abe M. (2015). Vehicle Handling Dynamics: Theory and Application.

[44] Emmanuel L.A., Christian C., Mbaocha C.C., Olubiwe M. (2019). Design of Two-Degree-Of-Freedom (2DOF) Steering Control for Automated Guided Vehicle. Int. J. Scienti. Eng. Sci..

